# Multi-functional magnesium alloys containing interstitial oxygen atoms

**DOI:** 10.1038/srep23184

**Published:** 2016-03-15

**Authors:** H. Kang, H. J. Choi, S. W. Kang, S. E. Shin, G. S. Choi, D. H. Bae

**Affiliations:** 1 Department of Materials Science and Engineering, Yonsei University, 134 Shinchon-dong Seodaemun-gu, Seoul, 120-749, Korea; 2 Department of Advanced Material Engineering, Kookmin University,77 Jeongneung-ro Seongbuk-gu, Seoul, 136-702, Korea; 3 Gangwon Research Institute Technology Research Center, 290 Daejeon-dong, Gangneung, 210-340 Korea

## Abstract

A new class of magnesium alloys has been developed by dissolving large amounts of oxygen atoms into a magnesium lattice (Mg-O alloys). The oxygen atoms are supplied by decomposing titanium dioxide nanoparticles in a magnesium melt at 720 °C; the titanium is then completely separated out from the magnesium melt after solidification. The dissolved oxygen atoms are located at the octahedral sites of magnesium, which expand the magnesium lattice. These alloys possess ionic and metallic bonding characteristics, providing outstanding mechanical and functional properties. A Mg-O-Al casting alloy made in this fashion shows superior mechanical performance, chemical resistance to corrosion, and thermal conductivity. Furthermore, a similar Mg-O-Zn wrought alloy shows high elongation to failure (>50%) at room temperature, because the alloy plastically deforms with only multiple slips in the sub-micrometer grains (<300 nm) surrounding the larger grains (~15 μm). The metal/non-metal interstitial alloys are expected to open a new paradigm in commercial alloy design.

One of the most interesting recent findings in the field of nanotechnology is the observation that nanoscale ceramic particles with extremely high surface energy can be decomposed in a metal melt, at a temperature much lower than their normal melting points^1^. This behavior allows the decomposed cationic and anionic elements to be separately alloyed. There is a large amount of thermodynamic data available regarding alloying cationic elements in metals. For example, aluminum decomposed from Al_2_O_3_ can be alloyed in magnesium metal. However, anionic elements such as carbon, nitrogen, or oxygen tend to form compounds instead of being dissolved in most metal systems because of the large difference in electronegativity between the anionic element and most metals^2^,^3^,^4^,^5^. One exception to this rule is found in steels (Fe-C alloy systems), where carbon is extensively soluble in liquid iron. Steels have been used over a large period in human history and are among the most technologically important alloys^6^ because they cover a wide range of the strength spectrum from low-yield to high-yield stress^7^. When carbon atoms do not have time to diffuse in large enough quantities to form compounds (Fe_3_C in Fe-C alloy systems), α-Fe (i.e., ferrite) becomes supersaturated with carbon atoms, forming a highly strained body-centered tetragonal phase (i.e. martensite); this behavior is a primary strengthening mechanism of steels^6^. This is an example of how non-metallic elements being alloyed in conventional metals can significantly alter the properties of the metal. However, metal-C/N/O alloy systems other than Fe-C alloys have not been the subject of much study.

Magnesium, which is of interest because of its low specific gravity (1.74 g/cm^3^), has significant potential for use in structural and functional components in the automotive, biomedical, and electronic industries^8^. The low density and, consequently, high strength-to-weight ratio of Mg provide a weight-saving potential, and its good processing capacities (e.g., good die-casting capability) lead to economic savings. Hence, the market for magnesium is rapidly growing and is expected to double within 4–5 years^9^,^10^.

Despite these advantages, both casting and wrought magnesium alloys in the industry suffer from corrosion and/or poor mechanical properties. Magnesium is highly reactive, particularly when magnesium is in contact with other metals, oxygen, and water. Based on thermodynamics, magnesium should completely react with oxygen, and its high intrinsic dissolution tendency accelerates corrosion in aqueous solutions. Furthermore, metal impurities and secondary phases in magnesium alloys stimulate local galvanic corrosion^11^,^12^. Meanwhile, because of the limited number of slip systems in the hexagonal close–packed crystal structure of magnesium, wrought magnesium alloys in sheet form provide limited deformability at ambient temperatures.

Substantial effort has been devoted to protect as-cast magnesium and its alloys from corrosion^13^ by producing a protective layer^14^ and/or by incorporation of a couple of alloying elements to control their microstructures^15^,^16^. However, a cost-efficient and environmentally friendly coating technique remains challenging to find. Certain trace impurities, such as iron, nickel, and copper, are reportedly notably harmful to the corrosion resistance of magnesium^17^. The addition of zirconium, which easily reacts with such impurities and effectively removes them, can improve the corrosion resistance of magnesium^18^. Aluminum has also been shown to improve the corrosion resistance of magnesium by increasing the amount of the *β*-phase^19^. The addition of rare earth elements, such as an yttrium, reduces the corrosion rates in dilute chloride solutions^20^. Despite the partial success of alloying approaches, the formation of passive films has not been realized.

Large–scale studies to improve the formability of wrought magnesium alloys have been conducted. In the conventional thermomechanical processes to fabricate sheets, a strong basal texture develops and remains essentially unchanged after the recrystallization annealing^21^,^22^,^23^,^24^. The sheets mostly deform via the <a>-slip in the basal plane, which is aligned along the rolling direction because the independent slip systems along the <*c*>-axis are difficult to activate. Mass flow along the transverse direction of the sheet occurs predominantly by mechanical twinning^24^. Thus, different deformation modes of the <a>-slip and mechanical twinning are simultaneously activated during deformation and do not allow an easy forming process. The contribution of mechanical twinning in the forming process can be mitigated by reducing the grain size of the sheet to approximately a few micrometers^25^. Such small grains can be developed using a special thermomechanical process, such as equal-channel angular pressing.

In this study, we propose a new alloying method that uses oxygen atoms in Mg metal. TiO_2_ nanoparticles are selected as an oxygen source because they are inexpensive and easily decomposed at approximately 400 °C, and the cationic titanium can be completely separated from the magnesium melt^26^. In the initial casting stage, TiO_2_ nanoparticles with high chemical potential energy are inserted into liquid Mg, and they decompose to O, Ti, and TiO^−^, as shown in Fig. 1 (stage 1). Most of the Ti and TiO^−^ sink and separate from the liquid (because of the relatively high specific gravity), which enables the oxygen atoms to be alloyed (stage 2). Because the amount of dissolved oxygen atoms are not sufficient to form magnesium oxides, they become supersaturated into the octahedral sites of Mg after solidification and create the Mg-O alloy (stage 3). Using the mother Mg-O alloy, a third element, such as aluminum or zinc, is alloyed to develop engineered magnesium alloys to elucidate the applicability of the Mg-O alloy as a casting material and a wrought sheet. The new Mg-O alloy can have significant potential for use in the magnesium industry, where high-performance, lightweight materials are in demand and would address critical issues in the research field of interstitial alloys.

To determine the position of oxygen atoms in a Mg-0.3 at. % O alloy, we observed the microstructure of the as-cast Mg-O alloy using high-resolution transmission electron microscopy (HRTEM) with energy-dispersive X-ray spectroscopy (EDS). Figure 2A reveals an EDS map of the as-cast Mg-O alloy, where Mg and O are presented in green and red, respectively. The detected oxygen atoms are randomly dispersed instead of being arranged with regular periodicity. Furthermore, the HRTEM image of the oxygen-containing area (marked region in Fig. 2A,B) shows typical Mg lattices instead of MgO. This result implies that oxygen atoms are dissolved in the Mg lattices without forming oxides. As revealed in the magnified image of the marked region (Fig. 2B), the Mg lattice is slightly expanded in most regions, with an estimated lattice spacing of 2.81 Å from the converted inverse fast Fourier transform (IFFT) image. This value is much larger than that of pure Mg (2.60 Å^27^). No body centered cubic structures (which are typical of MgO) are observed throughout the specimen. The diffraction pattern of the distorted lattice demonstrates the typical basal (0002) plane. Moiré fringes are also observed because of the overlap of pure Mg and expanded Mg-O lattices. These observations support the hypothesis that oxygen atoms occupy octahedral sites within the Mg rather than forming oxides, a behavior that leads to expanded lattice spacing.

The expanded lattice structure of the Mg-O alloy was further evaluated using X-ray diffraction (XRD). High-resolution XRD patterns of pure Mg and Mg-O alloy (Fig. 2C) show peaks for the (1011–0), (0002), and (1011–1) planes between 30 to 40° in 2θ; the peaks for the Mg-O alloy are located at 31.98° for (100), 34.29° for (0002), and 36.53° for the (1011–1) plane (in pure Mg, these values were 32.25°, 34.62°, and 36.84°, respectively). The peaks of all measured planes of the Mg-O alloy are shifted to lower angles compared to those of pure Mg, which indicates an increase of interplanar spacing as a result of lattice swelling. On the other hand, no peaks corresponding to magnesium oxides (29.3° and 38.5° in 2θ) are detected for either specimens in the XRD analysis.

In the X-ray photoelectron spectroscopy (XPS) depth profiles, the oxygen concentration in both Mg-O alloy and pure Mg was high (approximately 50 at. %) on the surface because of the surface oxide layer, whereas it steeply decreased with the increase in depth from the surface (Fig. S2 and Table S1). However, after removing a contaminated surface layer, a large amount of oxygen (0.3 at. %) was detected in the depth range of 15.2–18.2 μm in the Mg-O alloy, whereas the oxygen concentration in pure Mg was negligible (Fig. 2D). Furthermore, the comparison of the O 1s peaks of the Mg-O alloy and pure Mg, which were obtained at certain positions (0, 0.7, 3, and 6 μm from the surface), reveals that oxygen atoms were dissolved in the Mg-O alloys throughout the entire depth range, whereas the oxygen concentration decreased to ~0% below a depth of 3 μm in pure Mg (Fig. 3S). Based on these results, we conclude that oxygen atoms that are immersed in the magnesium liquid do not form oxides and are instead uniformly dispersed and supersaturated in the Mg lattices. Hence, they reveal no significant structural and chemical variation in the sample.

In this type of Mg-O alloy, the elastic modulus increases to a value approximately 10% greater than that of pure Mg (Table S2). It is hypothesized that the incorporation of nonmetal atoms in the interstitial spaces of magnesium may increase the Mg-O ionic bonding character and average Mg-Mg interatomic distance, which enhances the stiffness of magnesium^28^. The yield stress of the Mg-O alloy (~73 MPa) is also three times higher than that of pure Mg (Fig. 3 and Table S2). The lattice expansion together with the enhancement of stiffness and strength were also observed in a Mg-O alloy with 9 wt. % aluminum (Mg-O-9Al alloy) (Figs S4 and S5, respectively). According to the interstitial solid solution strengthening mechanism^29^,^30^,^31^, the introduction of interstitial oxygen atoms into a magnesium crystal produces a lattice dilation that typically gives rise to a spherically symmetric stress field. Thus, the interaction of dislocations with the resultant stress field gives rise to enhanced flow stress properties^32^.

For practical applications using this type of Mg-O alloy, the Mg-O-9Al alloy was studied further. As previously mentioned, this alloy exhibits enhanced mechanical properties (see supplementary text). In addition, when the magnesium alloy was exposed to an aqueous solution, it quickly eroded as a result of galvanic corrosion. This process occurred because of the potential difference between the matrix and the secondary phase^33^,^34^. This tendency is significantly mitigated when both phases contain oxygen atoms, as revealed in the Tafel plot (Fig. 4A). In particular, the corrosion potential increases by approximately 0.12 mV, and the current density is approximately 100 times lower in the Mg-9Al-O alloy than in the Mg-9Al alloy; the corrosion current density of the Mg-O-9Al alloy was 5.36 × 10^−7^A/cm^2^, while for the Mg-9Al alloy it was 2.33 × 10^−5^A/cm^2^. The result of electrochemical impedance spectroscopy (EIS) (Fig. S6) also supports that the oxygen containing alloys are covered with more uniform protective film that promotes the formation of passivation layers. Furthermore, the Mg-O-Al alloys exhibit better corrosion resistance than Mg-Al alloys in a more corrosive solution (3.5 wt.% NaCl solution, Fig. S7). The details of the corrosion behavior of these Mg-O alloys are available in the supplementary text.

To further protect the high intrinsic dissolution tendency of magnesium in aqueous solutions, a surface treatment, such as an anodizing or plating process, must be used. The environmentally friendly anodizing process is one of the most common and simple surface treatment processes used for this type of material. In general, large pores and a poor interface are generated near the interface of the matrix and the coating layer, when the Mg-9Al alloy is anodized by plasma electrolytic oxidation (PEO)^35^ (Fig. 4B). However, in the case of the Mg-O-9Al alloy, a clean interface is developed (Fig. 4C). During the PEO process, a significant amount of oxygen is supplied from both the solution and the alloy containing oxygen atoms, producing the tight Mg(Al)O oxide coating layer (see supplementary text). A comparison of the surface morphology of Mg-O-9Al (Fig. 5A) and Mg-9Al (Fig. 5B) alloys after the PEO treatment also reveals consistent results. The Mg-O-9Al alloy exhibits a relatively smooth surface, whereas the Mg-9Al alloy shows a large amount of pores, which can cause pitting in the corrosive solution.

The Mg-O alloy exhibits a superior capacity for plastic deformation. In general, Mg alloys with a hexagonal closed-packed structure have limited slip systems. Therefore, the plastic deformation of these materials can occur with the help of twinning activation at room temperature^36^. The typical flow curve of a Mg-2Zn sheet with an average grain size of 15 μm developed using conventional thermomechanical processes exhibits approximately 30% elongation to failure Fig. 6), and deformation twins are observed in the test sample (Fig. 6A). However, for a Mg-O alloy containing 2 wt. % Zn (Mg-O-2Zn alloy), fabricated using conventional rolling and annealing processes, the sheet exhibits significantly greater (>50%) elongation to failure without revealing the activation of twins (Fig. 6B). The flow curve of the Mg-O-2Zn alloy is also significantly different from that of the Mg-2Zn sheet. In the early stages of deformation, the yield-drop phenomenon is observed, and constant flow stress remains until 1.5% elongation is reached (clearly shown in the inset), similar to the typical yield-drop phenomenon observed in low-carbon steel^6^.

In general, during the hot-rolling process used to fabricate the sheet, many deformation twins with a low-angle twin boundary of 3.8° (tilted with respect to the matrix) are produced over an extensive area. The boundaries disappear when the sample is recrystallized during the annealing process, producing the final microstructure, as demonstrated in the Mg-2Zn sheet. In this range, dislocation activities along the *c*-axis are observed on the prismatic plane (Fig. S9). For the Mg-O-2Zn alloy sheet, however, many sub-boundaries form within the deformation twins as a result of the presence of accumulated oxygen atoms. These boundaries do not disappear, but instead develop as grain boundaries during the recrystallization process (see supplementary text, Fig. S8). A HRTEM image (Fig. 6C) of the deformed Mg-O-2Zn shows that the plastic deformation mainly occurs via non-basal slipping in sub-micrometer grains (<300 nm) that surround the larger grains (~15 μm), thereby providing extraordinary deformation capacity at room temperature that has not been observed in conventional Mg alloys.

Thermal conductivities of pure Mg, the Mg-O, Mg-9Al and Mg-O-9Al alloys were evaluated at room temperature as shown in Fig. 7. The alloys containing interstitial oxygen atoms exhibit much enhanced thermal conductivities (>20 W/mK enhanced) than their monolithic counterparts. A possible hypothesis to explain the high thermal conductivity of the Mg-O and Mg-O-9Al alloys is that the motion of interstitial oxygen atoms may help to transfer thermal energy in the preferential direction; the significant strain in the lattice surrounding the oxygen atoms may help them to easily jump to neighboring interstitial sites^37^, which dramatically changes the electronic population around the oxygen atoms^38^.

Excessively alloyed oxygen atoms in magnesium and in magnesium alloys increase the environmental resistance of these materials against mechanical loads or chemical corrosion, and significantly improve the plastic deformation and heat transfer capacities of these materials. Because oxygen atoms occupy the interstices of magnesium, ionic bonding character gradually dominates the system, which provides greater bonding energy. Changes in bonding energy alter the strength and formability of magnesium. Furthermore, the presence of oxygen atoms influences the corrosion properties of these alloys, leading to the formation of a protective layer via an anodization process. This allows anodizing particles, such as magnesium oxides, to be adsorbed homogeneously onto the magnesium surface. In addition, the solute oxygen atoms play an important role in thermal behavior and thermomechanical processes that govern the development of the superior alloy microstructures.

Here, we developed a new class of magnesium alloys that contain excessively dissolved oxygen atoms. The interstitial oxygen atoms, which do not form oxides and significantly expand the lattice spacing of magnesium, increase the environmental resistance of these materials against mechanical loads or chemical corrosion and improve the plastic deformation and heat transfer capacities of these materials. Further studies, particularly work involving the underlying physics regarding the atomic binding characters and their roles in the thermomechanical behaviors of these materials, are required and can offer interesting perspectives on how to achieve the exotic performances of metallic materials by atomic-level design.

## Materials and Methods

The Mg-O alloy was fabricated using a gravity casting method. Pure Mg was melted in a boron nitride-coated low carbon steel crucible under a dynamic SF_6_ + CO_2_ atmosphere, to which 1.0 vol. % TiO_2_ nanoparticles (with an average particle size of 50 nm) were put into a Mg melt at 720 °C. The alloy was held at this temperature for 30 min, and then the liquid material was slowly poured into a preheated rectangular steel mold (10 mm thickness). Pure Mg was also cast using the same method as a control.

Basic characteristics, including the lattice structure of the specimens, were analyzed using XRD (CN2301; Rigaku) with a Cu Kα radiation source (λ = 1.5405 Å). To evaluate the amount of oxygen atoms in the Mg-O alloy and pure Mg, the materials were examined using XPS (Thermo VG) with a monochromatic Al Kα source. Microstructural examination was also carried out using HRTEM (JEOL 2100FX) attached with an EDX spectrometer unit. Uniaxial tension and compression tests were carried out for the Mg-O alloy and pure Mg under a constant cross head speed with an initial strain rate of 1 × 10^−4^ s^−1^. The gage lengths of tension and compression were 10 and 5 mm, respectively. The elastic modulus was determined using an ultrasonic elastic constant measuring system (HKL-01-UEMT; Hankooklab). Electrochemical tests were performed with a conventional three-electrode cell in which a carbon plate was used as the counter electrode and a calomel electrode was used as the reference electrode. The thermal conductivity was determined by multiplying thermal diffusivity by the specific heat and density. The thermal diffusivity was measured by means of the laser flash technique (LFA447, NETZSCH, Germany), the specific heat was measured by differential scanning calorimeter (DSC, DSC8000, Perkin Elmer, USA), and the density of specimens was measured by Pycnometer (Ultrapycnometer 1000, Quantachrome Co. Ltd, USA).

## Additional Information

**How to cite this article**: Kang, H. *et al.* Multi-functional magnesium alloys containing interstitial oxygen atoms. *Sci. Rep.* **6**, 23184; doi: 10.1038/srep23184 (2016).

## Supplementary Material

Supplementary Information

## Figures and Tables

**Figure 1:**
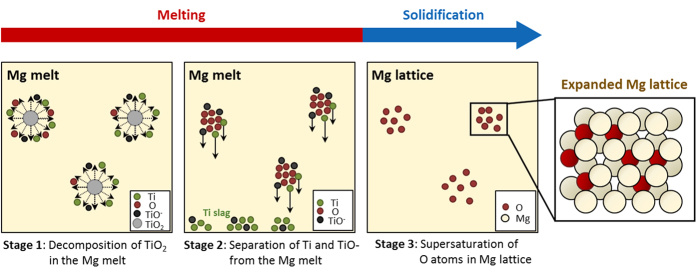
A schematic drawing of a fabrication process of the Mg-O alloy. TiO_2_ nanoparticles decompose to Ti, O and TiO^−^ in the Mg melt (stage 1); Ti and TiO^−^ separate out from the melt as slag and only O atoms are dispersed in the Mg melt (stage 2). Finally, O atoms fail to form oxides and are intercalated into the octahedral sites of Mg after solidification (stage 3).

**Figure 2:**
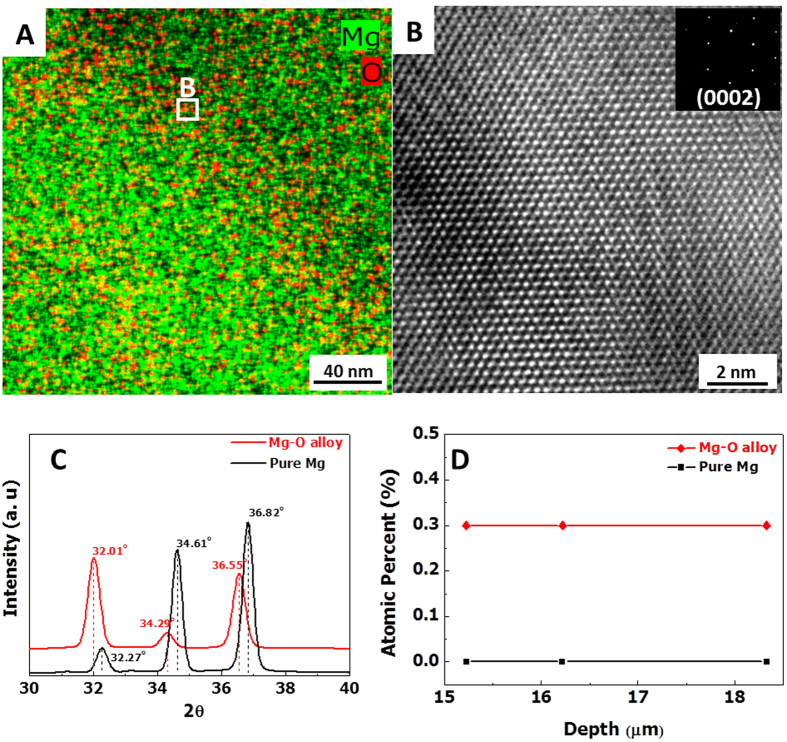
Structural and chemical analysis of the Mg-O alloy. (**A**) An EDS map of the as-cast Mg-O alloy. O atoms are randomly dispersed in the Mg matrix without forming magnesium oxides. (**B**) A HRTEM image of the marked region in (**A**) shows the expanded lattice structure of Mg by intercalation of O atoms where the image was obtained with the zone axis of [0002] as revealed in the selected area electron diffraction (SAED) pattern (inset). (**C**), XRD patterns also support the lattice expansion of Mg as O atoms occupy the interstices of Mg; peaks for the (100), (002), and (101) planes shift to lower angles for the Mg-O alloy and no oxides are detected in pure Mg and the Mg-O alloy. (**D**) Depth profiles of the oxygen concentration in pure Mg and the Mg-O alloy, as measured by XPS after removing the oxide layer (∼15.2 μm).

**Figure 3:**
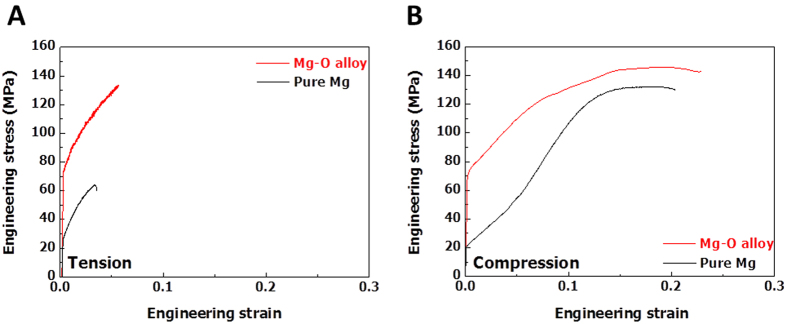
Mechanical properties of pure Mg and the Mg-O alloy. Engineering stress and strain relationships were obtained from uniaxial (**A)** tension and (**B**) compression tests with an initial strain rate of 10^−4^ s^−1^ at room temperature. The yield stress of the Mg-O alloy is more than three times greater than that of pure Mg without any decrease in its elongation to failure value.

**Figure 4:**
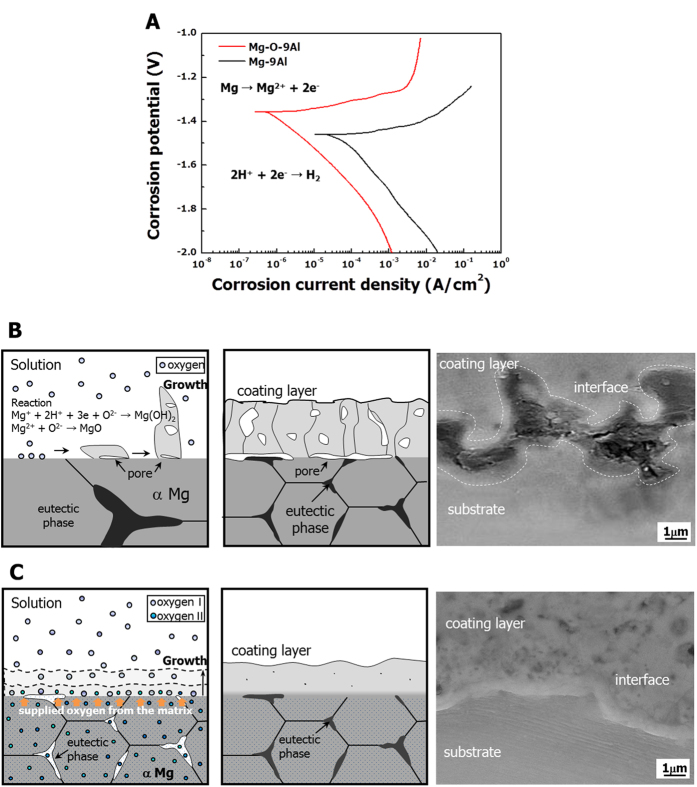
Corrosion behaviors of pure Mg and the Mg-O alloy. (**A**) Tafel plots of the Mg-O-9Al (in red) and Mg-9Al (in black) alloys in a 0.1 M NaCl solution at room temperature indicate that both the corrosion potential and corrosion current density of Mg are significantly enhanced by intercalation of O atoms. With respects to the corrosion rate, the Mg-O-9Al alloy shows a rate two orders of magnitude greater than that of the Mg-9Al alloy. The Mg-O-9Al and Mg-9Al alloys were coated using PEO, and the coated interfaces were observed using scanning electron microscopy (SEM). (**B**) The coated Mg-9Al alloy exhibits large pores between the matrix and the coating. (**C)** The coating layer in the Mg-O-9Al alloy was formed without pores in the interface as a result of the additional supply of oxygen from the Mg-O-9Al alloy.

**Figure 5:**
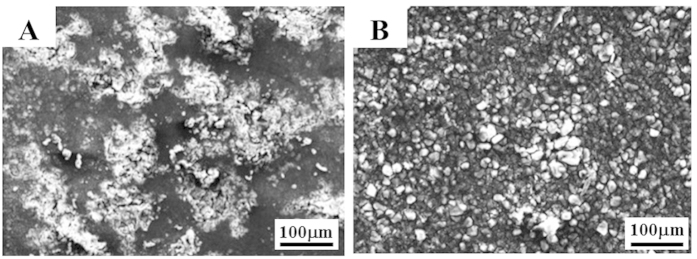
SEM images of the corroded regions of the anodized alloys: (**A**) Mg-9Al and (**B**). Mg-O-9Al alloys.

**Figure 6:**
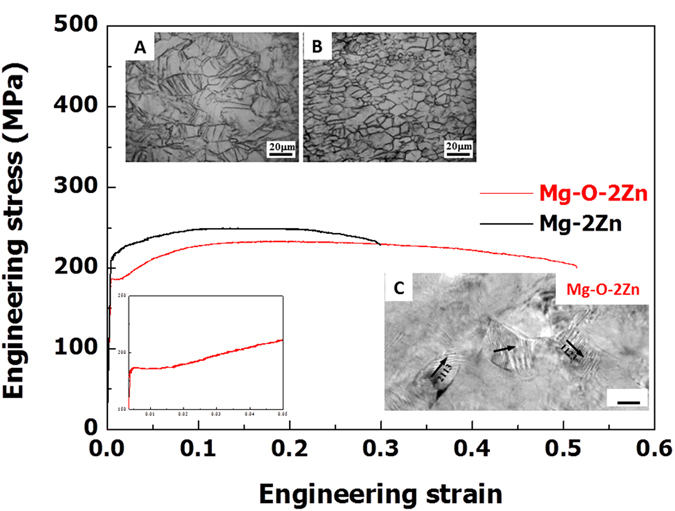
Tensile behaviors of Mg-2Zn and Mg-O-2Zn alloy sheets. The sheets, prepared by a conventional warm rolling process, were tested under uniaxial tension at an initial strain rate of 10^−4^ s^−1^ at room temperature. In the first stage of deformation for the Mg-O-2Zn sheet, yield drop phenomena occur (inset), as is commonly observed in Fe-C materials. (A) An optical microscopy (OM) image of the deformed Mg-2Zn sheet shows a development of many twins while (B) that of the deformed Mg-O-2Zn sheet exhibits a small number of twins. (C) A HRTEM image of the deformed Mg-O-2Zn shows that the plastic deformation mainly occurs via non-basal slipping in sub-micrometer grains (<300 nm) that surround the larger grains (~15 μm).

**Figure 7:**
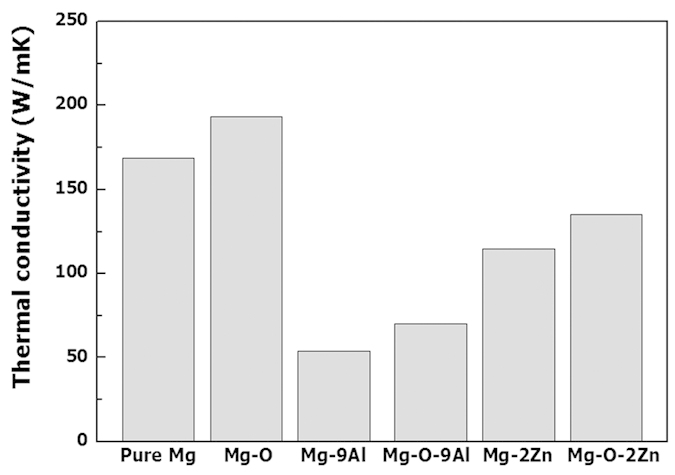
Thermal conductivities of pure Mg, Mg-O, Mg-9Al, Mg-O-9Al, Mg-2Zn and Mg-O-2Zn alloys. The thermal conductivity is significantly enhanced with the help of supersaturated O atoms.
