# Supplementary material for: Multi-functional magnesium alloys containing interstitial oxygen atoms
Source: Sci Rep. 2016 Mar 15;6:23184. doi: 10.1038/srep23184 (PMC4791639; doi:10.1038/srep23184)
Supplement: Supplementary Information [file srep23184-s1.doc]

**Multi-functional magnesium alloys containing interstitial oxygen atoms**

**H. Kang^1^, H. J. Choi^2^, S. W. Kang^1^, S. E. Shin^1^, G. S. Choi^3^ and D. H. Bae1[[1]](#footnote-2)***

1. *Department of Materials Science and Engineering, Yonsei University, 134 Shinchon-dong Seodaemun-gu, Seoul, 120-749, Korea*
2. *Department of Advanced Material Engineering, Kookmin University,77 Jeongneung-ro Seongbuk-gu, Seoul, 136-702, Korea*
3. *Gangwon Research Institute Technology Research Center, 290 Daejeon-dong, Gangneung, 210-340 Korea*

**A. XPS analysis**

Oxygen atoms have been reported to be soluble in pure Mg in the solid state (*1*). X-ray photoelectron spectroscopy (XPS) was used to analyze the amount of oxygen in the monolithic Mg and Mg-O casts fabricated in this study. The specimens were polished using a series of SiC polishing papers up to a maximum of 2000 grit. The XPS chamber was evacuated to a pressure of 6 × 10^-9^ mbar before the specimen was loaded. The analysis was conducted under an ultra-high vacuum pressure of 1 × 10^-9^ mb.

Survey scans were performed to collect broad low-energy spectra in the binding energy range of 1–1400 eV under an analyzer pass energy of 100 eV with a step size of 0.9 eV. Higher-resolution XPS spectra were then collected using an energy of 50 eV with a step size of 0.1 eV. High-energy-resolution spectra were collected for the elements assigned at each depth level, i.e., oxygen (O 1s) and magnesium (Mg 1s). All specimens were ion-sputtered using an argon gun operated at 1 keV to conduct in-depth profile analysis and to determine the thickness of the oxide film. For the removal of the surface of the specimen at the previously determined depth, an argon ion beam with a current density of approximately 2.8 μA/cm^2^ was rastered over a 2 × 2 mm area, yielding a sputter rate of approximately 0.27 nm/s; the beam was calibrated against a reference silicon oxide film on a silicon substrate. To compensate for the systematic error in the XPS analyzer, the energy of the C 1s peak (284.8 eV) was used as a standard binding energy.

The survey spectrum revealed the presence of Mg 1s, Mg 2s, Mg 2p, C 1s, O 1s, and O 2s peaks (Fig. S1). The two peaks at 25.59 and 530.60 eV correspond to the O 2s and O 1s core levels, respectively. Both of these peaks might be attributable to the oxygen decomposed from the titanium dioxide. However, such oxygen peaks can also arise from a MgO layer. An oxide layer can be produced during mechanical polishing or by the oxidation of a magnesium surface. In addition, carbon only exists on the topmost surface of the alloy.

The depth profiles of oxygen and magnesium in pure Mg (**A**) and in the Mg-O alloy (**B**) based on XPS analysis are exhibited in Fig. S2. The atomic percentages of oxygen and magnesium at certain depths are summarized in Table S1. In Figs. S2 **A** and **B**, the depth profiles in the range of 12 to 20 μm, respectively, are included for the identified low atomic concentration of oxygen. In Fig. S2 **A**, the atomic concentration of oxygen on the surface is high (approximately 50 at. %), whereas the concentration decreases steeply as the area below the surface is probed and saturates at increasing depths. The corresponding values are approximately 5 at. % at 10 μm and approximately 0.3 at. % at 15 μm below the surface. Oxygen atoms were detected at significantly higher fractions relative to magnesium atoms (approximately 26 at. %) on the surface, possibly due to surface oxidation during mechanical polishing. The concentration of magnesium increases as much as the concentration of oxygen decreases as sputtering is continued. In the pure Mg sample, the atomic concentration of oxygen is also substantial (approximately 50%) on the surface and then decreases rapidly, being almost absent at 3 μm below the surface as shown in Fig. S2 **B**. The concentration of magnesium in the pure Mg specimen increases steeply immediately below the surface and then saturates at 100 at. %. The Mg-O alloy was found to possess greater amounts of oxygen atoms compared to the pure Mg specimen, both on the surface and in the sub-surface region; the amount of oxygen resulting from the titanium dioxide addition is significant relative to the amount derived from the other substrate or from air. Furthermore, owing to the faster solidification rate at the surface, oxygen atoms migrate toward the surface for thermodynamic stabilization during solidification. The O 1s peaks of the Mg and Mg-O alloy samples were analyzed on the surface and at certain positions (0.7, 3, and 6 μm) below the surface and are shown in Fig. S3; the deconvolution of the peak at 0.7 μm below the surface is also shown in Fig. S3 **E**. The peaks in the spectra obtained from XPS profiles, as well as the angular-dependent measurements, are fitted to O 1s and Mg(OH)_2_: OH^–^ in Mg(OH)_2_ (531.6 ± 0.2 eV) and O 1s (529.4 ± 0.2 eV). The O to (OH^–^ in Mg(OH)_2_) atomic ratio value of 0.82 at the early stage of the profiling analysis suggests that some loosely bonded oxygen may be adsorbed on the oxide layer in the proximity of the surface; this type of oxygen is easily desorbed after the first sputtering cycle. The oxygen peaks in Fig. S3 **E** can be divided into O 1s and OH^–^ in the case of Mg(OH)_2_. Despite the low oxygen concentration shown in Fig. S3 **A**, the oxygen concentration increases with decreasing Mg(OH)_2_ concentration as the sputtering time is increased. During XPS analysis, oxygen is transformed into oxide ions because heat is readily applied to the sample by the X-ray beam. Although the same phenomenon occurs at the surface of pure Mg, for which the peak is divided into two components, the concentration of oxygen falls to 0% below a depth of 3 μm. On the basis of these results, it can be inferred that oxygen atoms can exist in and are soluble in magnesium.

**B. Lattice distortion**

X-ray diffraction (XRD) analysis was conducted on the Mg-O-9Al and Mg-9Al specimens to investigate the effect of interstitial oxygen atoms on the lattice structure of the magnesium matrix. Basic characteristics, including the lattice structure of the specimens, were analyzed using XRD on a diffractometer (Rigaku, CN2301) equipped with a Cu Kα radiation source (*λ* = 1.5405 Å). The XRD profiles were typically collected from 20 to 80° in 2*θ* (where *θ* is the Bragg angle) with a scan speed of 0.02 mm/s. To minimize the instrument error associated with shifting and broadening of a peak, a scan speed of 2 × 10^-4^ mm/s was used in the 2*θ* angle range of 30° to 40°. In Fig. S4 **A**, the peaks for the planes in the Mg-O-9Al alloy also shift to lower angles compared to those recorded for the Mg-9Al specimens. We magnified the peaks for the (100), (002), and (101) planes, as shown in Fig. S4 **B**. The angles of the peaks in the Mg-O-9Al alloy are 32.55° for the (100) plane, 34.73° for the (002) plane, and 37.02° for the (101) plane, whereas the monolithic Mg alloy (Mg-9Al alloy) shows the same peaks at 32.36°, 34.55°, and 36.84°, respectively. The shifting of peaks to lower angles indicates an increase of the associated interplanar spacings, which denotes lattice swelling due to the oxygen atoms.

**C. Mechanical properties**

To analyze the influence of interstitial oxygen atoms on the mechanical properties of the Mg-O alloy and pure Mg, we conducted tension and compression tests with an initial strain rate of 10^-4^ s^-1^ at room temperature. The corresponding flow curves are shown in Fig. 3. The materials alloyed with 9 wt. % Al were also tested and are shown in Fig. S5. The Vickers hardness and elastic modulus of the materials were analyzed, and the results are summarized in Table S2. The elastic modulus and the shear modulus were analyzed using an ultrasonic elastic constant measuring system (Hankooklab, HKL-01-UEMT). The elastic modulus and shear modulus were calculated on the basis of measurements of the velocity of the transverse and longitudinal waves (*2*). The values of the yield stress of the Mg-O alloy in both tests are higher than those of pure Mg (i.e., 73 MPa for Mg-O in both tests vs. 20 MPa for pure Mg in compression and 24 MPa in tension). In the Vickers hardness test, the values for the Mg-O alloys are twice as high as those of pure magnesium (41 Hv for the Mg-O alloy and 18 Hv for pure Mg). On the basis of these results, the distortion of the magnesium lattice by interstitial oxygen atoms is believed to affect the mechanical properties of the samples. The Mg-O alloy with the distorted lattice structure exhibits enhanced strength because the oxygen atoms occupying the interstices of the magnesium impede the movement of dislocations. The introduction of oxygen atoms into a magnesium crystal produces a lattice dilation that typically gives rise to a spherically symmetric stress field. The resultant stress field interacts with that of the dislocation, giving rise to a solute atom-dislocation interaction energy. Hence, the distorted lattice of the magnesium strengthens the matrix and leads to increased hardness.

**D. Corrosion resistance**

The materials were polished with up to 2000 grit abrasives and subsequently degreased with acetone and washed with distilled water. Next, the corrosion potential and current density were measured using a typical corrosion test. Electrochemical tests were performed with a conventional three-electrode cell in which a carbon plate was used as the counter electrode and a calomel electrode was used as the reference electrode. The tests were performed at 25 ± 1 °C in 0.1 M NaCl solution, where the circular testing area was 28.26 mm^2^.

The formation of a heterogeneous protection layer on the Mg-9Al alloy is an intrinsic property. In contrast, oxygen atoms contained in a typical Mg alloy contribute to the formation of a protective layer, such as a Mg(OH)_2_ film, when the alloy is exposed to aqueous conditions. For the Mg-O-9Al alloy, however, the oxygen atoms supplied to the surface lead to the formation of a homogenous Mg(OH)_2_ protection layer, and this layer strongly bonds to the surface.

The significantly improved corrosion resistance of the oxygen-containing alloys is caused by increased ionization energy. The ionization energy can be defined as the chemical free energy (Δ*G*) required to precipitate 0.1 mole of an ion on the surface of a metal (*3*). The chemical potential is given as
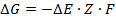
, where *Z*, *F*, and
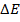
 are the atomic valence, the Faraday constant, and the equilibrium potential, respectively. According to the values of the equilibrium potentials of Mg and the Mg-O alloy, the chemical free energy values of pure magnesium and the Mg-O alloy in a 0.1 mole NaCl solution are 77.942 and 64.106 kcal/mol, respectively. Because of the oxygen atoms, the chemical energy is more stable, and the materials exhibit increased chemical resistance. Furthermore, the corrosion test was repeated under the relatively harsh condition (3.5 wt.% NaCl solution, Fig. S7) and the Mg-O-Al alloys exhibit superior corrosion resistance than Mg-Al alloys; the Mg-O-Al alloys exhibit higher corrosion potential (-1.465 V) as well as lower corrosion current density (4.289 μA/cm^2^) compared to Mg-Al alloys (-1.526 V for corrosion potential and 27.804 μA/cm^2^ for current density).

In Mg-Al-based alloys, galvanic corrosion occurs as a result of the potential difference between the Mg matrix and the alloy phases, as well as the relatively high potentials of the phases that contain Al. The oxygen atoms in the matrix modify the potential of the matrix so that the corrosion potential of the Mg matrix becomes similar to that of oxygen-containing eutectic phases. Due to the lack of an appreciable potential difference relative to other metals, galvanic corrosion cannot occur, and as a result the Mg matrix shows a low corrosion current density. Pure magnesium shows relatively low corrosion potential with a low corrosion current density, a phenomenon that is utilized in the coating process. For the Mg-O-9Al and Mg-9Al alloys, plates (30 × 50 × 2 mm^3^) were mechanically polished using 1000 grit emery paper and were subsequently rinsed with deionized water. Next, the plates were ultrasonically cleaned in ethanol and dried. The direct-current plasma electrolytic oxidation (DCPEO) process was adapted to coat the plates using a glass vessel container as an electrolyte cell under an applied current density of 50 mA/cm^2^. Stainless steel was selected as a counter electrode. A stirring and cold water jacket cooling system was installed to maintain the electrolyte at a constant temperature. The chemical compositions of the electrolytes used in this study are listed in Table S3.

**E. Wrought materials**

The effects of oxygen on the mechanical properties were investigated using a wrought alloy of Mg-O-2 wt. % Zn; Zn was added to enhance the ductility of the Mg-O alloy. The master Mg-O alloy was melted at 720 °C, and then 2 wt. % of Zn was added. The mixture was maintained at this temperature for 30 min. The melt was subsequently poured into a steel mold that was previously heated to 350 °C, and the thickness of the cast ingot was 10 mm. The ingot was homogenized at 300 °C for 4 h and was then hot-rolled to a thickness of 1 mm at this temperature with 25% reduction per pass. The rolled Mg-O-2Zn alloy sheet was annealed at 175 °C for 20 min. The Mg-2Zn alloy sheet was also fabricated via the same process for comparison.

The microstructures of the hot-rolled Mg-2Zn and Mg-O-2Zn alloy sheets exhibit numerous twins, most of which disappear after heat-treatment of the sheets at 175 °C for 20 min (Fig. S8). The average grain size of Mg-O-2Zn alloy sheet is approximately 4.28 μm and Mg-2Zn is approximately 15 μm. Furthermore, submicron grains can develop on non-basal planes and surround the larger grains. Non-basal slipping may occur as a result of compatibility stress at grain boundaries. The effects of compatibility on the activation of non-basal glide systems in Mg alloys are considered to be due to two separate grains of Mg under uniaxial tensile stress. The basal planes are oriented at 45° relative to the tensile axis in both grains but are tilted by 90° across the grain boundary. As only the basal slip system is activated, the two grains are deformed into a diamond shape. If the two deformed grains are to be bonded at a grain boundary, additional shear stresses (compatibility stresses) are needed, as well as the activation of non-basal slip systems (*5*). This result suggests that basal slip systems cannot operate near grain boundaries unless the non-basal glide systems become active, otherwise fracturing would occur along the grain boundaries (*6*). Because the density of mobile dislocations is expected to increase rapidly at this point, a clear stress-drop phenomenon appears, as exemplified in Fig. 5.

Figure S9 shows a high-resolution transmission electron microscopy (HRTEM) image of a 3%-deformed Mg-O-2Zn alloy sheet. The inset of the selected area diffraction (SAD) pattern indicates that the image was obtained on the zone axis of [101], i.e., the prismatic plane. The 〈113〉 dislocations lying on the {101} planes can be observed, and the {101}〈113〉 slip systems produce strain in the *a-* and *c*-axis directions, whereas the {0001}〈110〉 slip system cannot produce strain in the *c*-axis direction. In general, magnesium alloy sheets possess only two independent slip systems, basal slipping of 1/3<110> or 〈*a*〉-type dislocation, which are both associated with the primary deformation mode. Furthermore, the incorporation of non-basal slip of the 〈*a*〉 dislocations on the prismatic {100} plane offers only two additional independent slip modes. In contrast to the deformation mechanism of typical magnesium alloys, the Mg-O-2Zn alloy sheet is deformed by dislocations based on both the 〈*c*〉- and 〈*a*〉-slips. The activation of multiple slip systems may help the Mg-O-2Zn alloy sheet deform by more than 50%, even at room temperature. This hypothesis is also supported by the observation that dislocations are mostly generated around the Moiré fringes, where interstitial magnesium sites are occupied by oxygen atoms. The severely deformed lattice structure that results from the interstitial oxygen atoms helps to activate dislocations via multiple slip systems.

**F. Thermal conductivities**

The thermal conductivity of pure Mg and the Mg-O alloy determined by multiplying thermal diffusivity by the specific heat and density as follows:


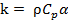
 (S1)

where *ρ* and *C_p_* and *α* are density, heat capacity, and thermal diffusivity of material, respectively.

The thermal diffusivity was measured by means of the laser flash analysis (LFA, LFA447, NETZSCH, Germany). The LFA method is that an energy pulse heats one side of a plane parallel specimen and then the temperature rise on the backside due to the energy input is time-dependent detected. The thermal diffusivity (*a*) is calculated from this temperature rise as follows (*7*):


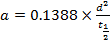
 (S2)

where d and t_1/2_ are the thickness of the specimen and the time to the half maximum, respectively. All specimens (diameter:
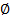
6 mm, thickness: ~2 mm) were polished up to 2000 grit and coated by graphite for the enhanced absorbance. The tests were carried out at the temperature; the values of the thermal diffusivity of Mg-O alloy are approximately 112 mm^2^/s at 25 ^o^C which is approximately 20 Wm^-1^K^-1^ higher than that of pure Mg.

The specific heat was measured by differential scanning calorimeter (DSC, DSC8000, Perkin Elmer, USA). The heat capacity (C_p_) is defined as (*8*):


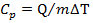
 (S3)

where *m* and ⊿*T* are mass of material and temperature change, respectively. *Q* is quantity of heat transferred energy. Since m and are ⊿*T* are unity, Q is a main parameter to change the value of C_p_. The value of pure Mg at 25 ^o^C is approximately 0.96 Jg^-1o^C^-1^, which is close to the literature values of pure Mg. The value of the Mg-O alloy at 25 ^o^C is 0.07 Jg^-1o^C^-1^ higher than that of pure Mg.

The density of specimens was measured by Pycnometer (Ultrapycnometer 1000, Quantachrome Co. Ltd, USA). The values of pure Mg and the Mg-O alloy at 25 ^o^C are 1.73 and 1.74 g/cm^3^, respectively. Furthermore, the densities of the Mg-9Al and Mg-O-9Al alloys were measured to be 1.81 and 1.81 g/cm^3^, respectively. The 0.3 wt. % oxygen atoms in the Mg-O alloy do not influence on the density.

**References**

1. Liao, J., Hotta, M. & Koshi, A. Effect of oxygen content on impact toughness of a fine-grained magnesium alloy. *Mater. Letters.* **65**, 2995 (2011).
2. Gupta, D. & Weinig, S. Interactions between interstitial and substitutional solutes in an H.C.P. lattice. *Acta. Mater.* **10**, 292 (1962).
3. Afifi, H. & Marzouk, S. Ultrasonic velocity and elastic moduli of heavy metal tellurite glasses. *Mater. Chem. Phys.* **80**, 517 (2003).
4. Jafarzadeh, K., Shahrabi, T. & Oskouei, A.A. Novel approach using EIS to study flow accelerated pitting corrosion of AA5083-H321 aluminum–magnesium alloy in NaCl solution. *J. Appl. Electochem.* **39,** 1725 (2009).
5. Anderson, T. L. Fracture mechanics : fundamentals and applications. (CRC Press, Boca Raton, ed. 2nd, 1995).
6. Koike, J. et al., The activity of non-basal slip system and dynamic recovery at room temperature in fine-grained AZ31B magnesium alloys. *Acta. Mater.* **51**, 2055 (2003).
7. Parker, W.J., Jenkins, R.J., Butler, C.P. & Abbott, G.L. Flash Method of Determining Thermal Diffusivity, Heat Capacity, and Thermal Conductivity. *J. Appl. Phys.* **32**, 9 (1679).
8. Kortz, J., Treichel, P., Townsend, J., & Treichel, D. Chemistry and Chemical reactivity 9th edition (Cengage Learning, Boston, 2015).

**Table S1**. Atomic percentages of Mg and O based on the XPS depth profiles.

| Depth (µm)  Material | | 0 | 0.7 | 1.5 | 3 | 6 | 9 | 12 | 15 | 18 |
| --- | --- | --- | --- | --- | --- | --- | --- | --- | --- | --- |
| Mg-O alloy | Mg 1s | 26.20 | 47.03 | 56.82 | 60.91 | 73.32 | 89.61 | 97.59 | 99.70 | 99.70 |
|  | O 1s | 73.80 | 52.97 | 43.18 | 39.09 | 26.68 | 10.38 | 2.50 | 0.30 | 0.30 |
| Pure Mg | Mg 1s | 27.33 | 53.18 | 100 | 100 | 100 | 100 | 100 | 100 | 100 |
|  | O 1s | 72.67 | 46.82 | 10.48 | 0 | 0 | 0 | 0 | 0 | 0 |

**Table S2**. Mechanical properties of the pure Mg and the Mg-9Al and the Mg-O alloys at room temperature.

| Materials | Poisson’s ratio | Shear modulus  (GPa) | Elastic modulus  (GPa) | Hardness  (Hv) | Compression | Tension | | |
| --- | --- | --- | --- | --- | --- | --- | --- | --- |
|  |  |  |  |  | Yield stress (MPa) | Yield stress  (MPa) | UTS (MPa) | Elongation to failure (%) |
| Pure Mg | 0.281 | 14.72 | 40.35 | 18 | 20 | 24 | 63 | 3 |
| Mg-O alloy | 0.301 | 16.41 | 44.04 | 41 | 73 | 73 | 133 | 5.6 |
| Mg-9Al alloy | 0.309 | 15.85 | 41.49 | 62 | 117 | 110 | 149 | 3 |
| Mg-O-9Al alloy | 0.332 | 16.88 | 44.56 | 77 | 159 | 143 | 210 | 7 |

**Table S3**. Electrolyte composition (M) used in the PEO process.

| Potassium  hydroxide | Potassium  fluoride | Potassium pyrophosphate |
| --- | --- | --- |
| 0.089 | 0.052 | 0.009 |

**Fig. S1**. XPS survey spectrum for the Mg-O alloy.

**A**

**B**

**Mg**

**Fig. S2**. Atomic percentages of Mg and O atoms in the Mg-O alloy (**A**) and pure Mg (**B**).

**A**

**B**

**C**

**D**

**E**

**Fig. S3**. The O 1s peaks of pure Mg and the Mg-O alloy at various depths: at the surface (A) and 0.7 µm (B), 3 µm (C), and 6 µm (D) below the surface and peak deconvolution of the O 1s peak at a depth of 0.7 µm (E).

**A**

**B**

**Fig. S4**. XRD patterns of the Mg-9Al and Mg-O-9Al alloys (**A**). Magnified XRD patterns from 30° to 40° with arrows identifying the top of each peak (**B**).

**A**

**B**

**Fig. S5**. Engineering stress and engineering strain relationship for the Mg-9Al and Mg-O-9Al alloys under tension (**A**) and compression (**B**) with an initial strain rate of 10^-4^ s^-1^ at room temperature.

**
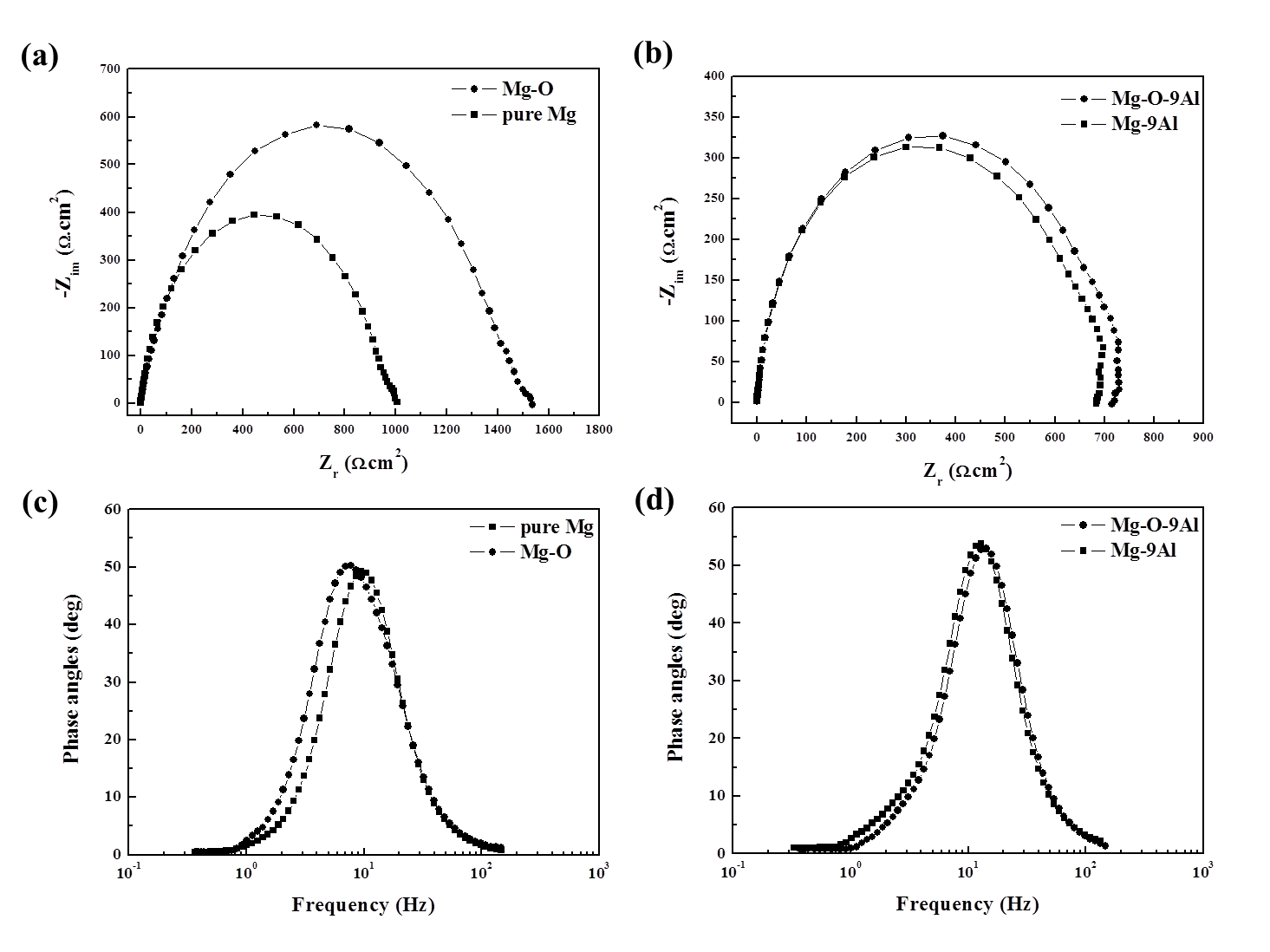
**

**B**

**A**

**D**

**C**

**Fig. S6.** Nyquist diagrams of **(A)** pure Mg with the Mg-O alloy and **(B)** Mg-O-9Al alloy with the Mg-9Al alloy. Bode spots of **(C)** pure Mg, Mg-O alloy and **(D)** Mg-9Al and Mg-O-9Al alloys.

**Fig. S7.** Tafel plots of the Mg-O-9Al (in red) and Mg-9Al (in black) alloys in a 3.5 wt.% NaCl solution at room temperature.


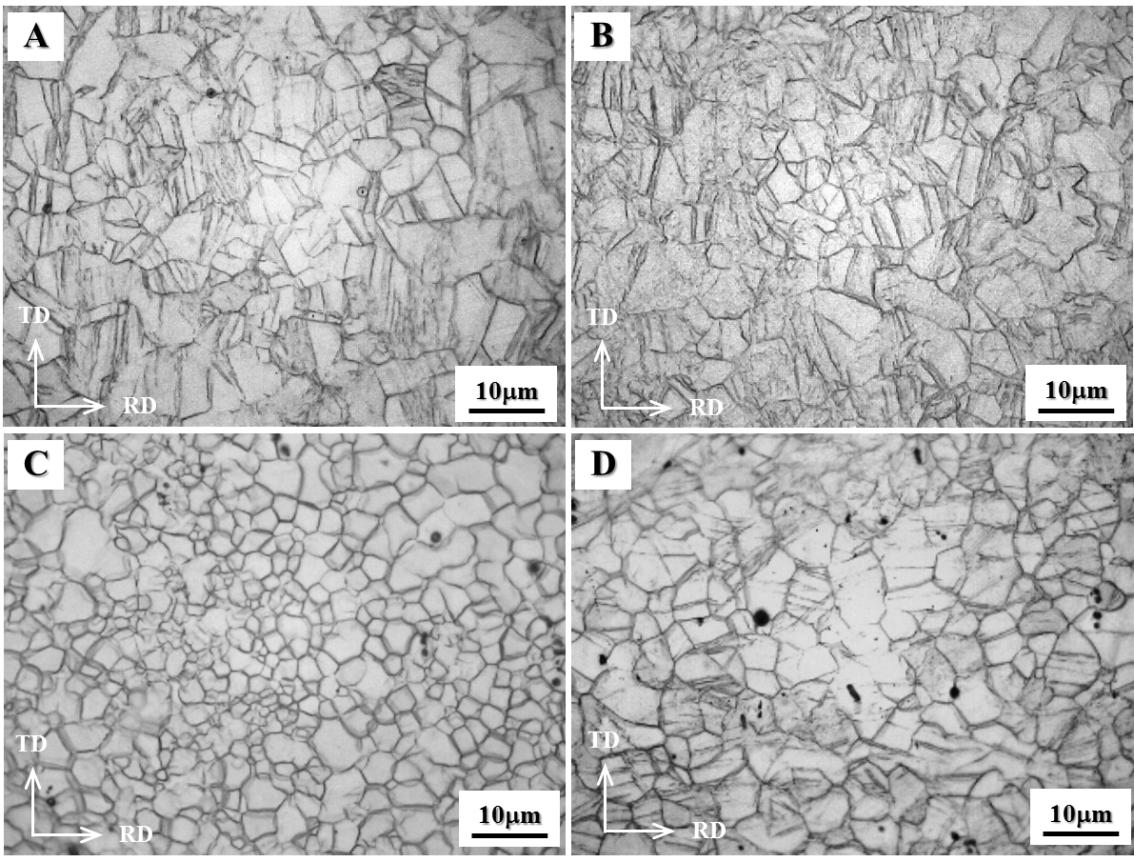


**Fig. S8.** Microstructures of the as-rolled sheets of the Mg-O-2Zn alloy (**A**) and the Mg-2Zn alloy (**B**) and heat-treated microstructures of the corresponding sheets at 175 °C for 20 min exhibited in (**C**) and (**D**), respectively.


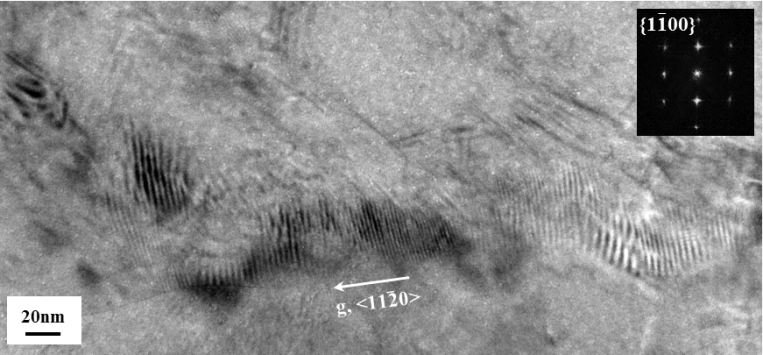


**Fig. S9**. HRTEM image of the <110> dislocations lying on {100} in the 3%-deformed Mg-O-2Zn sheet.

1. ^*^Corresponding author. Tel.: +82 2 2123 5831; fax: +82 2 312 5375

   *E-mail address*: [donghyun@yonsei.ac.kr](mailto:donghyun@yonsei.ac.kr) (D. H. Bae) [↑](#footnote-ref-2)
